# Psychiatric Sequelae Following Whiplash Injury: A Systematic Review

**DOI:** 10.3389/fpsyt.2022.814079

**Published:** 2022-04-22

**Authors:** Haidar Muhsen Al-Khazali, Håkan Ashina, Afrim Iljazi, Zainab Al-Sayegh, Richard B. Lipton, Messoud Ashina, Sait Ashina, Henrik W. Schytz

**Affiliations:** ^1^Danish Headache Center, Department of Neurology, Rigshospitalet Glostrup, Faculty of Health and Medical Sciences, University of Copenhagen, Copenhagen, Denmark; ^2^Department of Neurorehabilitation / Traumatic Brain Injury, Rigshospitalet Glostrup, Copenhagen, Denmark; ^3^Department of Neurology, Albert Einstein College of Medicine, New York, NY, United States; ^4^BIDMC Comprehensive Headache Center, Departments of Neurology and Anesthesia, Critical Care, and Pain Medicine, Beth Israel Deaconess Medical Center, Harvard Medical School, Boston, MA, United States

**Keywords:** anxiety, depression, post-traumatic stress disorder, sleep-disturbance, whiplash, headache

## Abstract

**Background:**

Anxiety, depression, post-traumatic stress disorder (PTSD), and sleep disturbance are reported following whiplash injury. However, the prevalence of these condition varies among studies. In this review, anxiety, depression, PTSD, and sleep disturbance will be referred as psychiatric outcomes.

**Methods:**

We performed a systematic literature search on PubMed and Embase (from database inception until March 20, 2021) to identify studies reporting on the relative frequency of these psychiatric outcomes. Three independent investigators screened titles, abstracts and full-texts. Studies including patients with whiplash injury and where the number of patients with whiplash and anxiety, depression, PTSD, or sleep disturbances could be extrapolated, were included. Furthermore, to be included, studies had to defined psychiatric outcomes in accordance with diagnostic criteria [i.e., Diagnostic and Statistical Manual of Mental Disorders (DSM) or the International Classification of Diseases (ICD)] or by use of a validated instrument with cut-off scores for assessing psychiatric symptoms. Quality rating was done using the Newcastle-Ottawa Scale (NOS) on the included studies. A protocol was registered with PROSPERO (CRD42021232037).

**Results:**

The literature search identified 5,068 citations, of which five articles were eligible for inclusion. The relative frequency of depressive symptoms following whiplash injury was 32.8% at 6 months, and 34.0% at 6–12 months. The relative frequency of PTSD symptoms after whiplash injury was 9.0–22.3% at 3 months, 15.8% at 6 months and 14.6–17.1% at 12 months. No studies evaluating the relative frequency of anxiety and sleep disturbances were eligible for inclusion.

**Discussion and Conclusion:**

Our results suggest that there are persistent psychiatric outcomes following whiplash trauma. However, we found considerable heterogeneity among the studies. Thus, we have focused on the most notable limitations of the included studies: 1) small sample sizes, 2) differences in enrollment criteria, 3) lack of control groups, 4) considerable variation in the method used for outcome assessment, 5) directionality of association is difficult to determine and 6) incomplete assessment of compensation factors. We highlight these methodological limitations and outline recommendations for future research. Since psychiatric outcomes are potentially modifiable, future studies should optimize and address the identified methodological limitations so psychiatric sequelae following whiplash injury may be prevented.

## Introduction

Whiplash injury is characterized by soft-tissue trauma to the cervical spine that follows sudden acceleration/deceleration movements of the head along with flexion/extension of the neck (1). The most common cause of this injury is rear-end car accidents (2, 3); epidemiologic studies have shown that a considerable proportion of those affected develop sequelae, such as neck pain and headache (4–6). Other sequelae are posited to include symptoms suggestive of depression, anxiety, post-traumatic stress disorder (PTSD), and sleep disturbances (7–9).

From a clinical standpoint, it is useful to categorize whiplash-related sequelae by their temporal relation to the injury. Some early-phase features begin within 3 months of the injury and include neck pain, headache, dizziness, and tinnitus (10). Psychiatric sequelae tend to develop after 3 to 6 months and might, in part, be attributed to the presence and persistence of early-phase sequelae (11). There is indeed some evidence to suggest that mental illness can develop as a consequence of chronic pain (12–14).

Here, we conduct a systematic review to examine the occurrence of depression, anxiety, PTSD, and sleep disturbances following whiplash injury. We also discuss methodologic aspects and outline directions for future research efforts.

## Methods

### Data Sources

This systematic review was registered on PROSPERO (identifier: CRD42021232037) and carried out in accordance with the guidelines for Preferred Reporting Items for Systematic Reviews and Meta-Analyses (PRISMA) reporting guideline (15). PubMed and Embase were searched from database inception (March 1952) to March 20, 2021, for observational studies that reported data on depression, anxiety, PTSD, and/or sleep disturbances following whiplash injury. The search term was “whiplash”; publications written in a language other than English were excluded. To expand the search, we reviewed the reference lists of the originally identified articles seeking additional studies.

### Study Selection

Three investigators (H.M.A., A.I., and Z.A.) independently performed the screening by titles, abstracts, and full texts. The inclusion and exclusion criteria are presented in Table 1. In brief, eligible studies had an observational design, included ≥30 participants, and reported data on the occurrence of anxiety, depression, PTSD, and/or sleep disturbances within 12 months of whiplash injury. Reviews, case series, and case reports were excluded.

**Table 1:** Inclusion and Exclusion criteria.

**Inclusion criteria**	**Exclusion criteria**
English language original articles	Abstract, conference proceedings or published articles in non-peer-reviewed journals
Sample-size of ≥30 persons with whiplash injury	Case reports, case series, review articles, animal studies
Random sample of whiplash injury patients with data reported on depression, anxiety, posttraumatic stress disorder and sleep disturbances 0–12 months after whiplash injury	Study populations selected based on the presence on specific symptoms, injuries or disorders other than whiplash injury
The author's case definition of whiplash injury was used to establish eligibility	Outcomes cannot be extracted properly
Clinic-based or population-based studies	
Psychiatric outcomes must be based on formal diagnosis from ICD or DSM or by validated instruments with cut-off scores.	

### Definitions of Psychiatric Outcomes

For the data-extraction, studies were eligible for inclusion if they defined psychiatric outcomes using established diagnostic criteria (i.e., DSM or ICD) or validated instrument with diagnostic cut-off scores for assessing psychiatric symptoms.

A formal diagnosis of anxiety (16–18), depression (19, 20), PTSD (21, 22) or sleep-disturbance (23, 24) was considered established if subjects met the criteria outlined in the Diagnostic and Statistical Manual of Mental Disorders (DSM) (edition 3 to 5) or the International Classification of Diseases (ICD) (edition 10). If anxiety, depression, PTSD or sleep-disturbance was evaluated by a validated instrument, it was deemed as a symptom rather than a diagnosis.

### Data Extraction and Quality Assessment

Data extraction was performed independently by three investigators (H.M.A., A.I. and Z.A.) using a standardized form and included information on the following: study design, age, sex, assessment method, whiplash-associated disorders (WAD) grade, time between whiplash and outcome assessment, number of subjects with whiplash injury, case definitions of anxiety, depression, PTSD and/or sleep-disturbance, number of subjects reporting anxiety, depression, PTSD and/or sleep disturbance, assessment method for the psychiatric outcomes. Any discrepancies in the data extraction were resolved between the three investigators, with two senior investigators (H.A. and S.A.) available to provide assistance. Study quality was assessed with a modified version of the Newcastle-Ottawa Scale (NOS) (25). The NOS consists of three domains (selection, comparability, and outcome) with a total of 9 items and a maximum obtainable score of 9. Two independent investigators (H.M.A. and A.I.) scored each study with any discrepancies resolved between the two investigators. Studies were scored as follows: 0–3=low quality; 4–6=medium quality; 7–9 =high quality.

## Results

**Figure 1:**
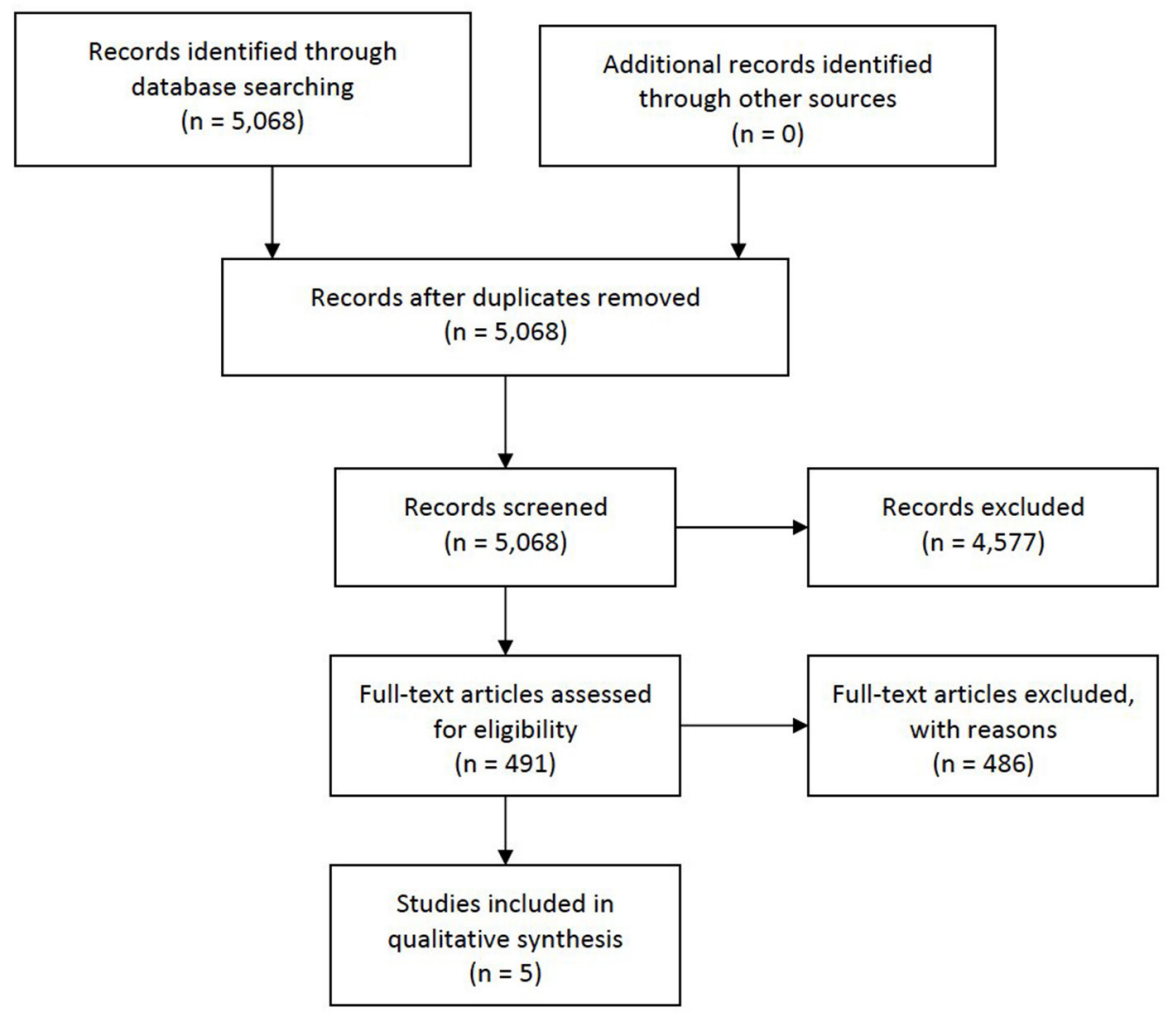
Flow diagram. Psychiatric outcomes following whiplash injury.

The database search yielded a total of 5,068 hits (Figure 1), and no additional articles were identified through a manual search of the refence lists of originally identified articles. We retrieved 491 articles for full-text screening and five were deemed eligible for inclusion (26–30). Of these, four studies reported data from a clinic-based sample (26–29), and one study provided population-based data (30).

All included studies reported data on the occurrence of symptoms suggestive of depression or PTSD using validated questionnaires with cut-off scores (26–30). None applied diagnostic criteria to classify cases in accordance with any iteration of the ICD or DSM. Furthermore, none of the included studies evaluated the relative frequency of anxiety and sleep disturbances. Study-level characteristics and proportions of depression and PTSD symptoms are reported in Table 2.

**Table 2:** Overview of the included observational studies.

**Psychiatric symptoms**	**Reference, publication year**	**Study type**	**WAD**	Whiplash^*^: number of subjects, age, sex (% female)	Controls^*^: type, number, age, sex (% female)	**Ascertainment of psychiatric status**	**Instrument name, type and cut-off (if applicable)**	**Time point post injury**	**Proportion subject with whiplash and the specified psychiatric symptoms**	**Proportion of controls with psychiatric symptoms**
Depression	Reme et al. (29)	Clinic-based, ED	1–2	*n* = 119, 39.0 ± 11.7 years, 69.0%	**Low back pain**, *n* = 387, 39.0 ± 10.6 years, 47.5%	Screening	HSCL-25, Questionnaire, 1.75	6–12 months	34.0%	**Low back pain** 29.0%
										**Disability pensioners** 46.0%
					**Disability pensioners**, *n* = 89, 49.0 ± 5.5 years, 65.1%					
	Cote et al. (30)	Population-based	NA	*n* = 175, 41.9 ± 12.4 years, 64.0%	**No Neck Injury after MVC**, *n* = 925^**^, 44.4 ± 13.0 years, 51.5%	Screening	CES-D, Questionnaire, ≥ 16	6 months	32.8%	19.3%
PTSD	Sterling et al. (27)	Clinic-based, ED	1–3	*n* = 155, 36.9 ± 12.8 years, 62.6%	No control group	Screening	PDS, Questionnaire, ≥ 0.50	3 months	22.3%	NA
								6 months	15.8%	NA
								12 months	17.1%	NA
	Hours et al. (26)	Clinic-based, ED, secondary and intensive care unit	1–2	*n* = 171, NA, 62.6%	**MVC with other mild injury**, *n* = 207, NA, 39.1%	Screening	PCL-S, Questionnaire, ≥ 44	12 months	14.6%	11.1%
	Hansen et al. (28)	Clinic-based, ED	1–3	*n* = 234, 37.5 ± 13.9 years, 61.5%	NA	Screening	HTQ and TSC, Questionnaire, HTQ ≥3 or TSC E2 criteria	3 months	9.0%	NA

* *Number of participants, age and sex is presented for the baseline assessment*.

** *Extracted from the exact numbers reported at each time point*.

*WAD, whiplash-associated disorders; ED, emergency department; n, number; NA, not available; HSCL-25, Hopkins Symptom Checklist-25; CES-D, Center for Epidemiologic Studies Depression Scale; DSM-4, Diagnostic and Statistical Manual of Mental Disorders 4. edition; PDS, Posttraumatic Diagnostic Scale; PCL-S, PTSD Checklist specific; HTQ, Harvard Trauma Questionnaire; TSC, Trauma Symptom Checklist*.

### Depression

#### Clinic-Based Studies

One retrospective emergency department based study investigated the symptoms of depression among whiplash patients (*n* = 119), low back pain patients (*n* = 387) and disability pensioners (*n* = 89) (29) (Table 2). The study screened patients using validated questionnaire (Hopkins Symptom Checklist-25 (HSCL-25). A cut-off ≥ 1.75 on the depression subscale was considered depression. The study showed that 34.0% screened positive for depressive symptoms 6–12 months following a whiplash injury (29). The corresponding results were 29.0% for those with low back pain and 46.0% for disability pensioners. The study did provide any statistical testing of differences among the groups.

#### Population-Based Studies

One population-based study reported on the symptoms of depression after neck injury in a motor vehicle accident (30) (Table 2). The population-based study provide data on patients with whiplash subjects (*n* = 175) and controls (*n* = 925). In fact, controls were defined as subjects with no neck injury after a motor vehicle crashes. To assess symptoms of depression a validated questionnaire (Center for Epidemiologic Studies Depression Scale (CES-D), cut-off at ≥ 16) was applied. The study screened for depressive symptoms 6 months after the injury and found that 32.8% of whiplash subjects and 19.3% of controls screened positive for these symptoms (30). The study showed no statistically significant difference in the depression symptomatology between the groups.

#### Study Assessment

The studies included in the review concerning depressive symptoms were of medium quality (29, 30) (Table 3). Both the clinic-based and population-based study had NOS scores of 6 points. In both studies, the relative frequency of depressive symptoms was numerically higher in persons with whiplash injury vs. controls, but statistically significant differences were not demonstrated.

**Table 3:** Quality assessment by Newcastle-Ottawa Scale (NOS).

**Author**	**Selection**				**Comparability**	**Outcome**			**Evaluation of study quality (number of star)**
	**Representativeness of the Whiplash sample**	**Selection of the control group**	**Ascertainment of exposure**	**Outcome absent at baseline**	**Assessment of comparability of exposed and unexposed groups**	**Assessment of outcome**	**Two or more follow-up assessments**	**Adequacy of follow-up**	
Reme et al. (29)	Somewhat representative^*^	Drawn from the same community^*^	No description	No	Study controls for co-morbidity^*^^*^	Screening^*^	Yes^*^	More than 20% lost	6
Cote et al. (30)	Somewhat representative^*^	Drawn from the same community^*^	No description	No	Study controls for co-morbidity^*^^*^	Screening^*^	Yes^*^	Small number lost (less than 20%)^*^	6
Hansen et al. (28)	Truly representative^*^	Drawn from the same community^*^	Physical examination^*^	No	No controls	Screening^*^	Yes^*^	More than 20% lost	5
Sterling et al. (27)	Truly representative^*^	Drawn from the same community^*^	Physical examination^*^	Yes^*^	No controls	Screening^*^	Yes^*^	Small number lost (less than 20%)	7
Hours et al. (26)	Truly representative^*^	Drawn from the same community^*^	Medical records^*^	No	No controls	Screening^*^	Yes^*^	More than 20% lost	5

***Quality scores**: Studies were scored as follows: 0–3=low quality; 4–6=medium quality; 7–9 =high quality*.

*Selection*

**(A)** Representativeness of the Exposed Cohort: Truly representative ^*^;

somewhat representative ^*^;

*selected group; no description of the derivation of the cohort*.

**(B) Selection of the Non-exposed Cohort**: Drawn from the same community as the exposed cohort ^*^;

*drawn from a different source; no description of the derivation of the non-exposed cohort*.

**(C) Ascertainment of Exposure**: Secure record (eg, medical records) ^*^;

structured interview ^*^;

*written self-report; no description*.

**(D) Demonstration that Outcome of Interest Was Not Present at Start of Study**: Yes ^*^;

*no*.

Comparability

**(E) Comparability of Cohorts on the Basis of the Design or Analysis:** Study controls for co-morbidity ^*^;

*study controls for any additional factors (age, sex, BMI); no controls*.

Outcome

**(F) Assessment of Outcome**: Independent or blind assessment ^*^;

record linkage ^*^;

*self-report; no description*.

**(G) Two or more follow-up time-points for Outcome report:** Yes ^*^;

*no*.

**(H) Adequacy of Follow-up of Cohorts**: Complete follow-up—all subjects accounted for ^*^;

subjects lost to follow-up unlikely to introduce bias—small number lost (less than 20% follow-up, or description provided of those lost) ^*^;

*follow-up rate more than 20% and no description of those lost; no statement*.

### Post-traumatic Stress Disorder

#### Clinic-Based Studies

Three clinic-based studies (26–28) investigated PTSD symptoms following whiplash injury (Table 2).

One prospective cohort study comprised 155 whiplash cases, evaluated symptoms of PTSD at 3 months, 6 months and 12 months following whiplash trauma (27). PTSD symptoms were screened by Posttraumatic Diagnostic Scale (PDS). The study found that 22.3% of whiplash subjects screened positive at 3 months, while this number decreased to 15.8% at 6 months and 17.1% at 12 months.

Another prospective cohort study included 173 subjects with “pure” whiplash injury and 207 with other mild injuries (control group). This study screened for PTSD symptoms using PTSD Checklist specific (PCL-S), cut-off score ≥ 44, 12 months post injury (26). At 12 months, PTSD symptoms were reported in 14.6% in the whiplash group, compared to 11.1% in the control group. However, no significant differences between groups for PTSD symptoms were found (26).

In a third study, Hansen et al. (28) performed a prospective cohort study with 234 whiplash subjects. Follow-up data was obtained at 3 months following a whiplash injury. Symptoms of PTSD were assessed by Harvard Trauma Questionnaire Part IV (HTQ) and Trauma Symptom Checklist (TSC) (28). The 3 months data revealed 9.0% screened positive for PTSD symptoms.

#### Study Assessment

The studies included in the review concerning PTSD symptoms, were considered to be of medium-to-high quality. Two study were deemed medium quality (NOS 5) (26, 28), while one study was deemed high quality (NOS 7) (27).

### Compensation

In the present review only one study provided information regarding seeking or receiving compensation among persons with whiplash injury (27). The study reported that submitting a compensation claim was statistically significantly associated with worsening of the PTSD and neck pain severity (27). Additionally, the most bothered subjects submitted a compensation claim shortly after the whiplash accident (27).

## Discussion

The data on the occurrence of psychiatric sequelae following whiplash injury are sparse. Indeed, no studies applied diagnostic criteria to examine the prevalence of these sequelae, and the available data were thus limited to observational studies that evaluated symptoms suggestive of depression or PTSD post-injury. The data showed depression- and PTSD symptoms to persist for up to 12 months after trauma. The proportions of psychiatric symptoms during the first year after whiplash were 32.7–34.0% for depressive symptoms and 9.0–22.3% for PTSD symptoms (Table 2). The wide distribution for PTSD symptoms may reflect variation in sample characteristics, the methods for assessing psychiatric symptoms and the timing of assessments following whiplash. Thus, the main finding from our review was the lack of studies and high-quality assessment, which renders it impossible to perform a reliable meta-analysis on the data. It is, therefore, of great importance to highlight the methodological limitations of current data on psychiatric symptoms following the whiplash injury and outline recommendations for future research efforts.

### Limitations

**Figure 2:**
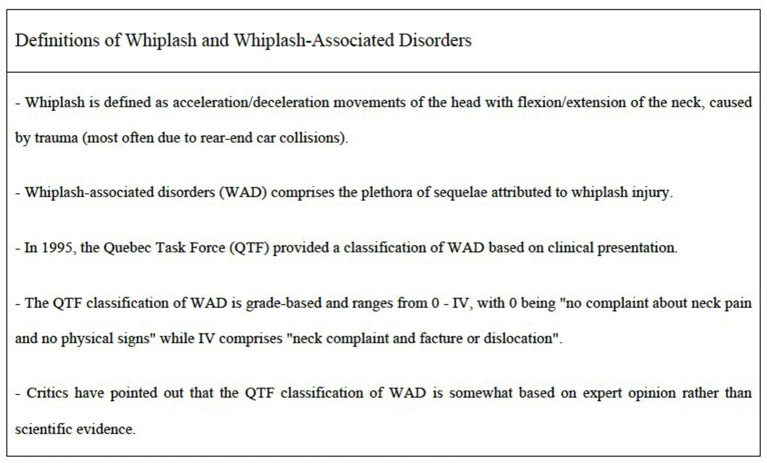
Commonly used definitions of whiplash and WAD (1).

First, whiplash studies concerning psychiatric outcomes had small samples sizes leading to wide confidence intervals and reduces power to detect differences. Many studies identify persons with whiplash at months to a year after a motor vehicle accident. Incomplete ascertainment of persons who were in a motor vehicle accident may lead to selection bias. Variation in follow-up time make it difficult to characterize the distribution of risk for psychiatric sequala at specific time points. Second, three out of four studies applied the Quebec Task Force (QTF) classification for WAD grading (27–29) (Figure 2). However, there was a high variation in the WAD grade of eligible study subjects, except that all included studies systematically excluded WAD grade 4. It merits emphasis, that only one of the included studies mentioned the reason behind the WAD grade 4 exclusion (28). They stated that since WAD grade 4 is characterized by having a fracture or dislocation, it is not exclusively a soft-tissue injury (28). Thus, the most affected whiplash subjects are not included, which limits the generalizability of our findings. None of the studies provided information needed for subgroup analysis nor investigated the association of WAD grade and the development of any psychiatric symptom. Third, the two clinic-based studies (26, 29) and one populations-based study (30) included controls with other traumas. Thus, none of the studies included healthy controls. Due to the lack of healthy controls, it is difficult to assess if whiplash subjects are more likely to develop psychiatric symptoms compared to other groups and healthy controls. Fourth, all included studies used validated screening instruments. However, the applied instruments deviated from formal diagnosis, which may explain higher proportions in the analyzed studies. Furthermore, some of the validated instruments (such as HSCL, HTQ and TSC) include questions concerning general pain, neck pain and headache. In whiplash samples it could be recommend eliminating these pain-related questions to avoid overestimating rates of psychiatric symptoms. Fifth, the included studies used questionnaires that relied on symptom reporting rather than diagnostic interviews and application of accepted diagnostic criteria (DSM or ICD criteria). Even though the studies used validated tools with cut-off scores to assess psychiatric symptoms, these tools have not been validated against a clinical gold standard diagnosis based on DSM or ICD criteria. Questionnaires are more feasible in large scale studies than semi-structured interviews by a trained diagnostician, but the later approach is more rigorous. Sixth, it is difficult to determine the direction of effects even though these are longitudinal studies. People with psychiatric symptoms may be more prone to experience accidents. Sleep disorders, for example are well-known to contribute to the risk of accidents (31, 32) and anxiety is well-known to interfere with performance (33, 34). The post-traumatic headache following a whiplash injury might resemble those of primary headache disorders, such as tension-type (TTH) headache and migraine (35, 36). In fact, evidence suggests correlations of post-traumatic headache, TTH and migraine with psychiatric comorbidities (e.g., depression, anxiety, PTSD, sleep-disturbance, and cognitive disfunction) (37–39). There are various plausible scenarios for these correlations. Psychiatric comorbidities may be risk factors for the development of whiplash-related complications, such as a chronic post-traumatic headache. Otherwise, psychiatric comorbidities are consequences of the whiplash injury, rather than to the post-traumatic headache. Finally, they may be associated to each other by both scenarios. In continuation, some may wonder if personality traits are predictors to psychiatric sequelae followed by whiplash injury. This has been addressed by two systematic reviews, where both articles concluded that personality traits had no association with the whiplash prognosis (40, 41). Seventh, compensation- and litigation-related factors might also influence the whiplash prognosis among studies. In the present review only one study reported the compensation status of whiplash patients (27). The nature of psychiatric and physical outcomes following whiplash injury have led to controversy over the determination of their cause and appropriate compensation (42–44). A study by Cassidy et al. reported a 28.0 % reduction in the number of claims after changing from tort to no-fault compensation system (45). However, by their analysis they also concluded that whiplash subjects recover faster if compensation for pain and suffering is not available. This is supported by other studies which have found that minimal intervention in the acute period provides recovery (46, 47). In fact, some studies claim ongoing compensation process affects the subject's quality of life negatively (48, 49). It may also be assumed that whiplash subjects with a high degree of pain may be compensated more frequently. However, one study did not find any statistical association between the level of pain and compensation status (50). Instead, it found correlation between the relatively high levels of mental distress (not defined) and financial compensation (50).

### Future Perspectives

Future research on psychiatric sequelae following whiplash injury is warranted and should be designed to properly avoid and address the above-mentioned methodological limitations. Future studies should apply standardized case definitions of both whiplash injury and psychiatric symptoms. In addition to self-reports and screenings at the initial assessment, gold standard diagnostic interviews or validated instruments with empirical cut-scores based on DSM or ICD criteria are required to provide a better and reliable estimate of the burden of the psychiatric sequelae over the course of the follow-up period. These instruments should be appropriate for a whiplash population and ideally validated in the population of intended use. The confounding effects WAD grade, pre-injury psychiatric history, perhaps including family history, need to be evaluated in prospective studies. To determine if psychiatric disorders are risk factors for whiplash injury, it might be important to investigate following: 1) Are rates of psychiatric symptoms or diagnosis higher in persons with whiplash than the general population and if so, at what time point following the injury? 2) Are rates of psychiatric symptoms or diagnosis higher in persons with whiplash than in uninjured or injured persons who were in a motor vehicle accident of equivalent severity? 3) Is the whiplash injury in the causal pathway linking motor vehicle accident to psychiatric symptoms or diagnosis?

It is important to answer these questions because psychiatric outcomes are potentially modifiable. Finally, future research should investigate whether early interventions and preventative modalities contribute to the recovery of WAD. Thus, it is importance to address, understand and deal with psychiatric symptom form the very beginning of their manifestation.

## Conclusions

This review has qualitatively summarized the relative frequency of depressive and PTSD symptoms following whiplash injury. The estimated relative frequencies of depressive symptoms were found to be 32.7–34.0%, while 9.0–22.3% for PTSD symptoms within the first-year post injury. Substantial heterogeneity was evident among the studies. Moreover, we shed light on a significant number of limitations within the whiplash literature. Due to these limitations, it is almost impossible to determine frequencies, causality, and the direction of causality. Further studies utilizing standardized definitions and validated instruments are much warranted to address and prevent the development of chronic WAD and related sequelae.

## Data Availability Statement

The original contributions presented in the study are included in the article/supplementary material, further inquiries can be directed to the corresponding author.

## Author Contributions

HMA, HA, and SA conceived and designed (including search strategies) the review. HMA did the literature search with AI and ZA. HMA wrote the first and subsequent drafts of the manuscript. HA, AI, ZA, MA, SA, RL, and HS participated in critical revision and writing of the article. All authors have seen and approved the final version.

## Funding

MA was supported by the Lundbeck Foundation Professor Grant (R310- 2018-3711).

## Conflict of Interest

The authors declare that the research was conducted in the absence of any commercial or financial relationships that could be construed as a potential conflict of interest.

## Publisher's Note

All claims expressed in this article are solely those of the authors and do not necessarily represent those of their affiliated organizations, or those of the publisher, the editors and the reviewers. Any product that may be evaluated in this article, or claim that may be made by its manufacturer, is not guaranteed or endorsed by the publisher.

## Abbreviations

PTSD: post-traumatic stress disorder
DSM: Diagnostic and Statistical Manual of Mental Disorders
ICD: International Classification of Diseases
WAD: Whiplash-associated disorders
QTF: Quebec Task Force
HSCL-25: Hopkins Symptom Checklist-25
CES-D: Center for Epidemiologic Studies Depression Scale
PCL-S: PTSD Checklist Scale
HTQ: Harvard Trauma Questionnaire
TSC: Trauma Symptom Checklist
PDS: Posttraumatic Diagnostic Scale
TTH: Tension-Type Headache.
